# A novel prognostic signature for hepatocellular carcinoma based on SUMOylation-related genes

**DOI:** 10.1038/s41598-023-38197-4

**Published:** 2023-07-11

**Authors:** Jianping Wang, Peipei Cong, Zhipeng Jin, Lingli Liu, Dongxu Sun, Wenjing Zhu, Guangjun Shi

**Affiliations:** 1 Department of Hepatobiliary Surgery, Qingdao Municipal Hospital, Qingdao, China; 2 Qingdao Municipal Hospital, Qingdao, China; 3 Clinical Research Center, Qingdao Municipal Hospital, Qingdao, China

**Keywords:** Oncogenes, Computational models

## Abstract

SUMOylation (SUMO modification) has been confirmed to play an essential role in the progression of various malignancies. As the value of SUMOylation-related genes (SRGs) in prognosis prediction of hepatocellular carcinoma (HCC) has not been explored, we aim to construct an HCC SRGs signature. RNA sequencing was utilized to identify differentially expressed SRGs. The 87 identified genes were used in Univariate Cox regression analysis and the Least Absolute Shrinkage and Selection Operator (LASSO) analysis to build a signature. The accuracy of the model was validated by the ICGC and GEO datasets. The GSEA revealed that the risk score was associated with common cancer-related pathways. The ssGSEA showed that NK cells in the high-risk group were significantly reduced. The sensitivities of anti-cancer drugs confirmed the sensitivity of the high-risk group to sorafenib was lower. Further, our cohort showed that risk scores were correlated with advanced grade and vascular invasion (VI). Finally, the results of H&E staining and immunohistochemistry of Ki67 showed that higher-risk patients are more malignant.

## Introduction

Hepatocellular carcinoma (HCC) is one of the most commonly diagnosed cancer types worldwide^1^. This severe disease, which accounts for the majority of the primary liver cancer cases, has high mortality and is typically developed from chronic hepatitis and cirrhosis^2,3^. Despite the enormous advances achieved in diagnostic techniques, the overall survival period of patients with HCC is shorter due to late disease identification and diagnosis^4^. Over the years, traditional treatments for HCC patients showed poor clinical effects^5^. VI is a significant factor affecting prognosis in HCC^6^. Another study also reported that patients with VI, compared to those without evidence of VI, showed a shorter median survival^7^. Hence, VI was included in our study as an important clinical indicator.

SUMOylation is a protein modification pathway that regulates various biological processes, including cell division, signal transduction, DNA repair, and cell metabolism^8^. SUMOylation consists of a three-step enzymatic reaction, including activation, coupling, and ligation^9^. Accumulating evidence has shown that many cancers have significantly enhanced SUMOylation dynamics^10^. Thus, SUMOylation can be viewed as a global mechanism that increases the stability and robustness of complex signaling pathways, which, if unchecked or spuriously activated, can exert disastrous consequences for cells^10^. The abnormal expression of SUMOylation might be a cause of tumor progression and could thus serve as a novel marker^11^. Currently, due to the ease of access to public databases, a growing number of signatures have been discovered that predict the patients’ prognosis, whereas no SUMOylation-related risk signature has been identified in HCC patients.

In the present research, we screened out SRGs related to prognosis in HCC, and analyzed the TCGA database by Lasso Cox regression to develop a model. Moreover, the predictive accuracy of the risk feature was tested in ICGC, GEO cohort, and our cohort. The research might provide a new method for the clinical treatment of HCC.

## Results

### Identification of HCC prognosis-associated DEGs

**Figure 1:**
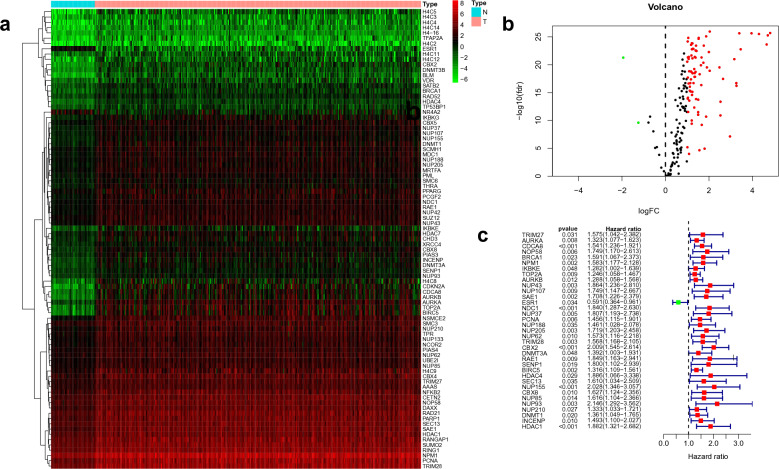
Identification of HCC prognosis-related DEGs. (**a**) Heatmap of the 87 identified SRGs; (**b**) Volcano map of the 87 identified SRGs; (**c**) Forest plot of the univariate Cox regression results.

We obtained the expression data of the mRNA sequences of 50 normal tissue and 374 HCC tissue samples by searching the TCGA database. Differentially expressed SRGs in tumor and normal samples were filtrated by the limma package in R. We found that 2 and 85 SRGs were significantly down-regulated and up-regulated, respectively. The information of these findings was displayed in the heat map and volcano map, depicted in Fig. 1a,b. Thirty-five SRGs were closely related to HCC prognosis by univariate Cox regression analysis (Fig. 1c).

### Identification of subtypes in HCC

**Figure 2:**
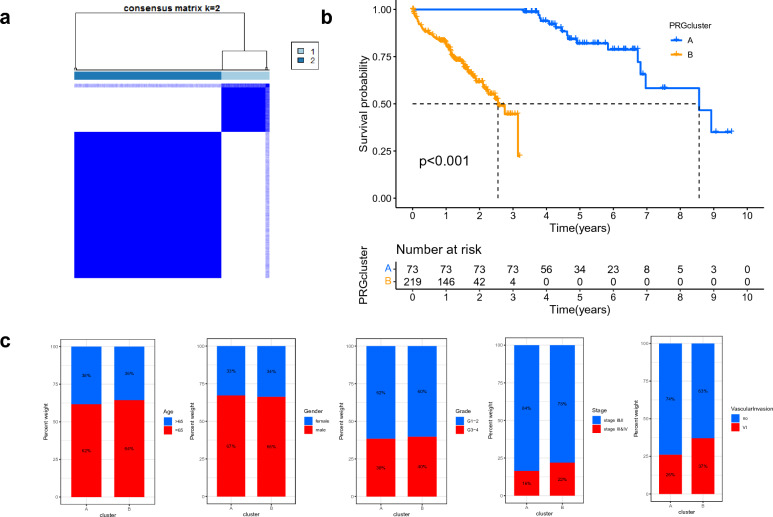
SRG subtypes and clinical characteristics of two distinct subtypes. (**a**) Consensus matrix heat-map defining two clusters (k = 2); (**b**) Comparison of OS among two subtypes; (**c**) Comparison of clinical information between the fibrosis-related clusters.

The result of the consensus clustering algorithm revealed that k = 2 seemed to be a desirable choice to divide the whole cohort into A (n = 73) and B (n = 219) (Fig. 2a). The K–M curve revealed that patients in subtype A had a more prolonged OS than patients in subtype B (Fig. 2b). There was no significant difference in clinical features between the two subtypes (Fig. 2c).

### Construction of a SRGs-based prognostic model

**Figure 3:**
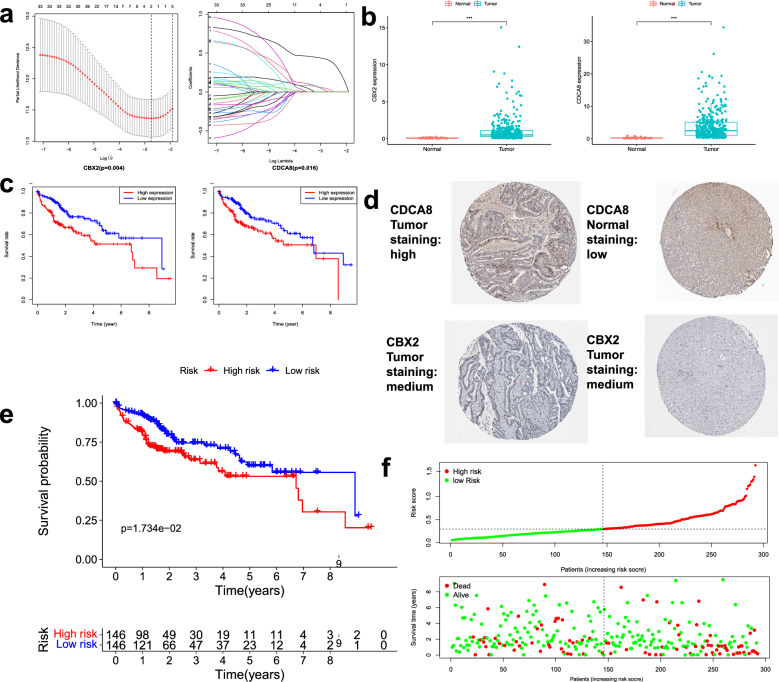
Development of a novel prognostic signature. (**a**) Partial likelihood deviance versus log (lambda) drawn. (**b**) Expression levels of two genes in HCC and normal samples; (**c**) The K–M curve of model genes; (**d**) Immunohistochemical staining protein level from the HPA database; (**e**) The patients in the low-risk groups have significantly longer OS outcomes than those in the high-risk groups; (**f**) Risk score distribution in HCC patients, overall survival and survival status of HCC patients in the TCGA database.

Subsequently, the 35 genes were analyzed by Lasso regression analysis for building a risk signature. 2 genes (CDCA8 and CBX2) remained with the minimum partial likelihood deviance, of which two were risk factors (Fig. 3a). Further research showed that the mRNA expression in the two model genes in normal tissue was significantly lower than that in the tumor tissue samples (Fig. 3b). In addition, the high expression levels of the two SRGs indicated a low survival rate in the K–M curve (Fig. 3c). Immunohistochemical (IHC) staining protein level obtained from the HPA database showed the results of protein expression were consistent with transcription levels (Fig. 3d). This prognostic signature was developed to calculate the risk score by the following formula: Risk score = (0.03166 × expressions of CDCA8) + (0.40426 × expressions of CBX2). Then, based on the median risk score, HCC patients were divided into two groups in the TCGA cohort: high-risk (n = 146) and low-risk (n = 146). We used K–M curves to compare the difference in the overall survival between the two groups. The high-risk HCC patients, compared to the low-risk group, had a worse 5-year survival probability (Fig. 3e). Patient’s survival time was presented as a scatter plot, and the risk scores were ranked in ascending order, revealing that the high-risk patients had a worse prognosis (Fig. 3f).

### Evaluating the prognostic value

**Figure 4:**
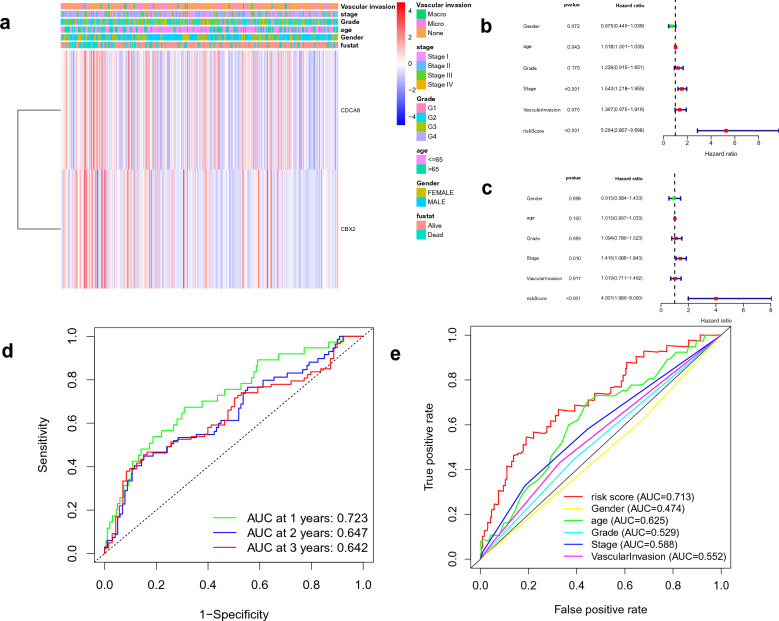
Evaluation of the prognostic value. (**a**) A heat-map of two model genes in five clinical indicators in TCGA; (**b**) prognostic effect analysis of risk score and clinical features in HCC with univariate and multivariate Cox regression analysis; (**c**) Time-dependent ROC curves for predicting 1-, 2-, and 3-year OS of TCGA cohort; (**d**) The ROC analysis of the risk score and other prognostic clinical features in HCC.

Initially, the two model genes expression and clinical characters were shown on the heat map (Fig. 4a). Then, univariate Cox regression analyses demonstrated that age, stage, and risk score could predict the prognosis of HCC patients (Fig. 4b). Risk score and stage were found to be independent prognostic factors by multivariate Cox regression analysis (Fig. 4c). Furthermore, the time-dependent ROC curves revealed that AUC values of 1-, 2-, and 3-year in the TCGA cohort were 0.723, 0.647, and 0.642, respectively. These results suggested that the prognostic model was effective in accurately predicting the survival time (Fig. 4d). To assess the clinical significance, we developed multi-factor ROC curves, which showed that the risk score (AUC 0.713) was better for predicting the survival time of HCC patients than those of other clinical factors (Fig. 4e).

### Validation of the prediction ability by the ICGC database

**Figure 5:**
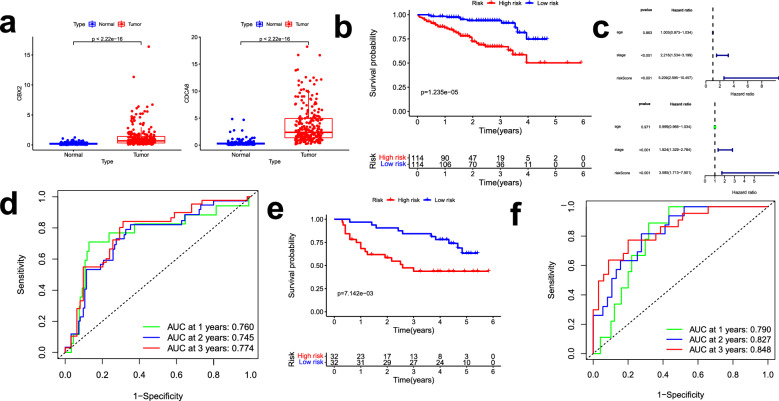
Validation of the prediction ability in the external database. (**a**) The expression level of the two genes in the ICGC cohort; (**b**) K–M analysis of the high- and low-risk groups among the HCC samples in the ICGC; (**c**) The univariate and multivariate Cox regression analysis of the risk score; (**d**) Time-dependent ROC curves for predicting 1-, 2-, and 3-year OS of ICGC cohort; (**e**) K–M analysis in the GSE116174; (**f**) Time-dependent ROC curves of GSE116174 cohort.

We utilized the ICGC and GSE116174 cohorts to validate the accuracy of the prognostic signature. First, the expression levels of two genes in the tumor tissues were found to be also higher than in the normal tissues (Fig. 5a). Next, based on the computational formula, the HCC patients in the ICGC database were also divided into two sets. The results showed that the patients in the low-risk group had a better survival time (Fig. 5b). The univariate and multivariate analyses suggested that risk score and stage could be used as independent indexes for prognosis prediction (Fig. 5c). In addition, the AUC values of the time-dependent ROC curves (1-, 2-, and 3-year) were 0.760, 0.745, and 0.774, correspondingly (Fig. 5d). In the GSE116174 cohort, we found that we found that higher-risk score meant worse OS (Fig. 5e). And 1-, 2- and 3-year AUC of OS were 0.790, 0.827 and 0.848, which showed that the risk signature met the criteria for prognosis prediction (Fig. 5f).

### Correlations between the risk model and the clinical factors

The association between risk score and clinical factors was explored. Our results showed that the risk score was related to the grade, stage, and VI of patients (Supplementary Fig. 1a). The patients with advanced stage, higher grade and VI had higher risk scores in the TCGA database. Meanwhile, the risk score was related to the stage in the ICGC cohort (Supplementary Fig. 1b).

### Nomogram construction

**Figure 6:**
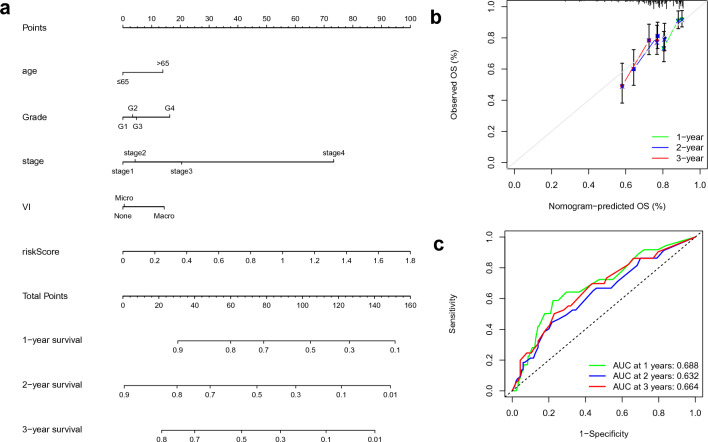
Nomogram construction. (**a**) Nomogram for predicting the 1-, 2-, and 3-year OS in HCC patients; (**b**) Calibration curves of nomogram; (**c**) The 1-, 2-, and 3-year ROC of Nomogram.

To apply the prognostic model for the prediction of the survival time of HCC patients, we further combined the age, stage, grade, and VI with the risk score to build the 1-, 2-, and 3-year OS prediction nomograms. Based on the nomogram, we could build an average patient score to determine patients’ OS (Fig. 6a). In addition, and the calibration diagrams indicated that the nomogram had an excellent performance (Fig. 6b). The AUC of the nomogram model revealed the satisfactory accuracy for 1-, 2- and 3-year OS (0.688, 0.632, 0.664) (Fig. 6c).

### Pathways correlated with the risk score

To explore the signaling pathway underlying the SRGs model, we conducted GSEA. The results revealed that SUMOylation and several common tumor-related pathways, such as cell cycle, neurotrophin signaling pathway, pathways in cancer, base excision repair, MAPK, VEGF, and P53 signaling pathway, were significantly enriched in the high-risk patients (Supplementary Fig. 2).

### Correlation between the prognostic model and tumor immune micro-environment

The ssGSEA showed that patients with high-risk scores had a significantly higher level of immune cell infiltration, including Macrophages, aDCs, Tfh, Treg, and Th2 cells, but lower proportions of Natural killer (NK) cells (Supplementary Fig. 3a). Interestingly, immune-related functional pathways, such as the score of Type-II IFN response, CCR, Checkpoint, MHC class I, APC co-stimulation, were different between the low- and high-risk group in the TCGA cohort (Supplementary Fig. 3c). In the ICGC cohort, the ssGSEA demonstrated the result of immune cell infiltration (e.g., B cells, Neutrophils, NK cells and Th2 cells) and immune-related functional pathways (e.g., Type-I IFN Response and Type-II IFN Response) (Supplementary Fig. 3b,d). In conclusion, NK cells and Type-II IFN Response of high- and low-risk had statistically significant differences in TCGA and ICGC.

### Drug susceptibility analysis

**Figure 7:**
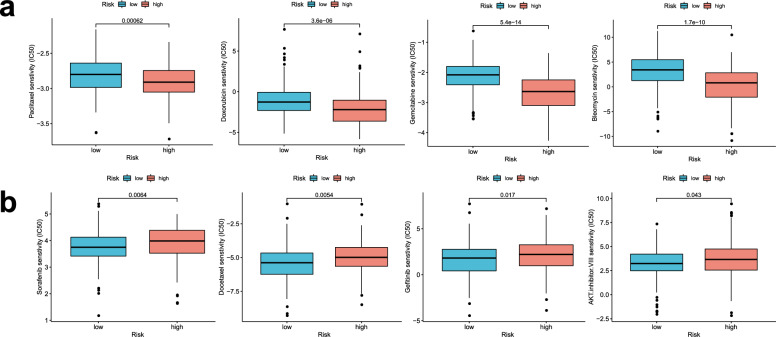
Sensitivity of the different risk groups to chemotherapy. (**a**) Lower IC50 values in high-risk patients. (**b**) Higher IC50 values in high-risk patients.

Eight common chemotherapy drugs were selected to analyze to examine the sensitivity of different risk groups to chemotherapy. We analyzed that the high-risk patients’ scores had lower IC50 values for paclitaxel, gemcitabine, doxorubicin, bleomycin (Fig. 7a), whereas the IC50 values of chemotherapeutics, such as sorafenib, gefitinib, docetaxel, and AKT inhibitor VIII were significantly lower in the patients with low-risk scores (Fig. 7b). To sum up, the aforementioned results showed that the risk scores were associated to drug sensitivity.

### Verification of clinical tissue samples

**Figure 8:**
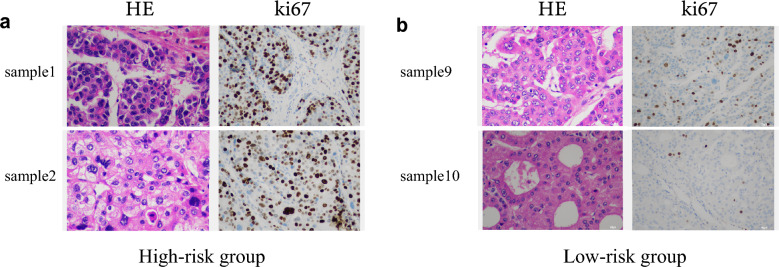
HE staining of tumor tissues and immunohistochemistry was used to detect the expression of Ki67 in HCC tissues. (**a**) High-risk patient (Samples 1, 2); (**b**) low-risk patient (Sample 9, 10).

Further, we validated the accuracy of the signature in our cohort from Qingdao Municipal Hospital. First, we standardized the expression level and obtained the risk score by the formula: Risk score = (0.03166 × relative expression of CDCA8) + (0.40426 × relative expression of CBX2). The median risk score was utilized as a cut-off value to classify patients into a high-risk (n = 5) or a low-risk group (n = 5). The risk scores of these ten patients and their clinical information are listed in Table 1. Next, we analyzed the relationship between the risk scores and clinical factors, which displayed that the risk score was associated with vascular invasion and tumor grade by Fisher Chi-square tests (Table 2). And results of H&E staining and immunohistochemistry in samples 1, 2, and samples 9, 10 are exhibited in Fig. 8a,b.

**Table 1:** Clinical parameters of 10 HCC from clinical patients.

Sample	Grade	Stage	VI	Risk score	Risk
1	G3	Stage III	Invasion	0.43592	High
2	G3	Stage II	Invasion	0.378746328	High
3	G4	Stage III	Invasion	0.148692076	High
4	G3	Stage II	Invasion	0.14270414	High
5	G3	Stage I	Invasion	0.114524725	High
6	G2	Stage I	No	0.088184136	Low
7	G3	Stage I	No	0.083630926	Low
8	G2	Stage IV	Invasion	0.07453907	Low
9	G2	Stage IV	No	0.07453907	Low
10	G1	Stage I	No	0.058986327	Low

**Table 2:** The fisher Chi-square tests.

Characteristics		Risk		P value
		High	Low	
Grade	1–2	0	4	0.0238
	3–4	5	1	
Stage	I&II	3	3	1
	III&IV	2	2	
VI	No	0	4	0.0238
	Invasion	5	1

## Discussion

HCC is a deadly disease with a very low 5-year survival rate^12^. Therefore, it is critically important to build a reliable and effective prognostic model for patients with HCC. Numerous medical studies in the past have shown that SUMOylation is closely related to tumorigenesis, metastasis, and proliferation and is significantly upregulated in most tumors^13,14^. In this study, we first screened 187 DEGs in the TCGA cohort. We analyzed the relationship between DEGs and HCC patients’ prognosis by univariate Cox analysis. We identified two specific molecular subtypes based on the expression of prognosis-related genes in our study. Then, we used LASSO regression analysis to develop a two-gene SUMOylation-related predictive model in the TCGA database and tested its accuracy using the ICGC and GEO databases. Further, we found that the high-risk patients had a worse prognosis compared with the low-risk patients. Finally, we estimated the performance of the risk model in the following aspects: clinical characteristics, GSEA, tumor immune micro-environment, and chemotherapeutic susceptibility to several drugs. The aforementioned results revealed that this prognostic signature had good clinical guidance significance and could be used to predict patient prognosis.

SUMOylation is necessary to maintain genome integrity and regulate gene expression and intracellular signaling^15^. Both SUMOylation and de-SUMOylation were involved in the pathogenesis of cancer^16^. This most likely represented a subtle homeostasis of the SUMOylation state of proteins involved in DNA repair, cell division, and cell signaling in normal cells, as well as dysregulation in cancer cells^17^. In addition, the interaction between ubiquitination-like and other reversible post-translational modifications (phosphorylation, acetylation, and ubiquitination) is a process that occurs repeatedly in vivo, because the dependence of one modification on the other greatly expands the specificity and regulatory potential of each reversible post-translational modification^18^. However, the relationship between SRGs and the development of HCC is still unclear. In prevent study, the SUMOylation-related gene (SUMO-2 and SAE1) were up-regulated in HCC, and high levels correlated with a worse survival time^19^.

The risk model included two genes (CDCA8 and CBX2). Two genes were over-expressed in HCC tissues, which was associated with a low survival rate. The previous study demonstrated HCC cell progression was inhibited by the knockdown of CDCA8. This process was achieved by restoring the ATF3 tumor suppressor and restraining the AKT signaling pathway^20^. Molecular targeted therapy of CDCA8 might be an effective systemic approach to prevent tumor recurrence by eliminating cancer stem cells and cancer cells^20^. CBX (Chromobox Homolog 2) in the PRC1 complex SUMOylates CETN2 at an unknown residue with SUMO2,3^21^. The knockdown of CBX2 expression in HCC cells increased HCC cell apoptosis and suppressed HCC cell proliferation^22^.

We constructed a risk model to provide further, more effective guidance on clinical diagnosis and treatment. Our study showed that the high-risk group was correlated with vascular invasion, advanced stage, and higher grade. In the present study, vascular invasion was a very important clinical index^23^. Both micro-invasion and macro-invasion were correlated with poor survival^24^. Pawlik et al.^25^ reported that patients with vascular invasion had significantly shorter median survival time compared to patients with no evidence of vascular invasion. We found that high-risk groups were more likely to develop vascular invasion in TCGA and our cohort. These results could guild the clinical works. For instance, high-risk groups need early surgical treatment to prevent vascular invasion. In addition, high-risk patients were more likely to experience tumor recurrence.

The GSEA indicated that a high-risk score was significantly associated with some common HCC potential pathways (e.g., SUMOylaton, neurotrophin signaling pathway, cell cycle, and so on). And the association of most of these pathways with the occurrence and therapy of HCC has been previously validated. For example, it was becoming clear that cancers exhibited substantially enhanced SUMOylation dynamics^10^. Loss of normal cell cycle control was an important beginning of the tumor. Cancer cells accumulate alterations leading to unscheduled proliferation and genomic instability^26^. Mesencephalic astrocyte-derived neurotrophic factor (MANF) levels were associated with the status of liver cirrhosis, advanced stage, and tumor size^27^. We forecast that the activation of these pathways might be the reason for high-risk patients with poor survival. In recent studies, the application of the immune micro-environment has been used as a novel anti-cancer therapy^28^. The results for NK cell and IFN response II had statistical significance in our study. NK cell was the main anti-tumor cell in the liver, which could affect other immune cells’ anti-tumor behavior^29^. Previous studies had observed that the SUMOylation inhibitor enhanced the proportions of activated NK cells in vivo treatment^30^. The results showed that the immune infiltration of NK cells in the high-risk group was significantly reduced, which could explain the reason for the high-risk group with the poor prognosis. IFN response plays a crucial role in promoting host anti-tumor immunity and is considered to be pivotal component in the cancer-elimination phase of cancer immunosurveillance^31^. In this study, the sensitivity of the high-risk group to sorafenib was lower than that of the low-risk, whereas higher gemcitabine sensitivity was observed in the high-risk patients. Therefore, high-risk patients resistant to sorafenib can be treated with gemcitabine, which may achieve better results.

We developed an SRGs-related risk model and tested the accuracy of the risk model using several approaches. Subsequently, we further explored the possible mechanism and pathways involved. Certainly, the risk model had limitations. First, only external cohorts and the Qingdao Municipal Hospital cohort (only ten samples) were included in this study. Second, no further functional in vivo or in vitro experiments were conducted to reveal the potential mechanisms of the gene model.

## Materials and methods

**Figure 9:**
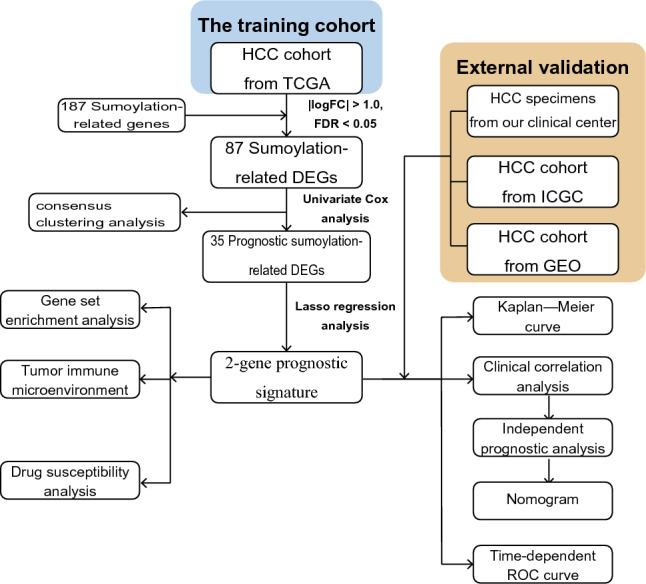
The flow chart.

The flow chart was presented in Fig. 9.

### Patients and HCC specimens

HCC tissues were acquired from 10 HCC patients who received an operation at Qingdao Municipal Hospital (Qingdao, Shandong, China) in 2020 which were frozen for western blotting. Meanwhile, the clinical information of each patient was documented in detail. Each patient signed informed consent. The research had been approved by the Ethics Committee of Qingdao Municipal Hospital. All assays were consistent with the Declaration of Helsinki regulations.

### RNA extraction and qRT-PCR

After tissue grinding, total RNA was extracted with TRIzol reagent (Tiangen, China) according to the manufacturer’s protocol. cDNAs were obtained from total RNAs by using PrimeScript RT reagent Kit (TaKaRa, Japan). The real-time PCR (qRT-PCR) experiment was performed using TB Green Premix Ex Taq II (TaKaRa, Japan). The expression levels were normalized with GAPDH. The primer sequences used in this study are displayed in Table 3. The reaction parameters included a denaturation program (30 s at 95 °C), followed by an amplification and quantification program over 40 cycles (5 s at 95 °C and 34 s at 60 °C). Each sample was tested in triplicates, and each sample underwent a melting curve analysis to check for the specificity of amplification. The expression level was determined as a ratio between the model genes and the internal control GAPDH in the same mRNA sample and calculated by the comparative CT method. The expression levels of model genes were calculated by the 2 − δδCt method.

**Table 3:** Primer sequences for two genes and GAPDH.

Primer name	Primer sequence
CDCA8 forward	AGCAGGACAGTTGGCAGCAG
CDCA8 reverse	AGTCCCACTGACCACCTCCC
CBX2 forward	GCGGCTGGTCCTCCAAACAT
CBX2 reverse	TGGCAGTGAGCTTCCTTGGC
GAPDH forward	GACCTGACCTGCCGTCTA
GAPDH reverse	AGGAGTGGGTGTCGCTGT

### Data acquisition

We downloaded the data of the mRNA expression and the clinical information of the included HCC patients from the TCGA, ICGC, and GEO databases. Duplicate and missing data in databases were deleted. A total number of 187 SUMOylation-related genes were downloaded by gene sets “REACTOME_SUMOYLATION” from the GSEA website^32^. The data downloaded from TCGA, ICGC, and GEO (GSE116174) databases was freely publicly available. The clinical information from public databases were shown in Table 4.

**Table 4:** The clinical characteristic information of the HCC patients in TCGA and ICGC.

Characteristics	TCGA (%)	ICGC (%)
Number	292	228
Age		
< 65	176 (60.27)	81 (35.53)
≥ 65	116 (39.73)	147 (64.47)
Gender		
Male	194 (66.44)	NA
Female	98 (33.56)	NA
Survival status		
Alive	205 (70.21)	185 (81.14)
Dead	87(29.79)	43(18.86)
Stage		
Stage I&II	232 (79.45)	141 (61.84)
Stage III&IV	60 (20.55)	87 (38.16)
Histological grade		
G1–2	177 (60.62)	NA
G3–4	115 (39.38)	NA
Vascular invasion		
None	192 (65.75)	NA
Micro or macro	100 (34.25)	NA

### Prognosis-related DEG screening

First, we evaluated the differentially expressed SRGs from the TCGA database by the “limma” package in R software (version 4.0.2, https://www.R-project.org/)^33^, based on the following standard: |log_2_ Fold Change |> 1.0 and FDR < 0.05. The results were shown by heat map and volcano map. Next, the univariate Cox analysis was utilized to determine the prognostic SRGs.

### Consensus clustering analysis

We used the R package “ConsensusClusterPlus” to establish a novel according to the expression of the prognosis-related gene^34^. We further explored the OS of different molecular subtypes by R package “survival” and “survminer”. We next evaluated the correlations between molecular subtypes and clinical characteristics.

### Prognostic signature establishment

The Lasso regression analysis was employed to develop a formula using the “glmnet” package in R software. The signature genes were further analyzed by expression levels and survival rates in TCGA databases. The protein expression level of candidate genes was tested in the Human Protein Atlas database (HPA)^35^. Finally, the survival time of the high- and low-risk groups was established by the K-M curve.

### Signature accuracy validation

First, univariate and multivariate Cox analyses were employed to validate the independent prognostic value of the risk score. Next, time-dependent receiver operating characteristic (ROC) curve analysis was applied to evaluate the accuracy of the signature by the “timeROC” package^36^. A C-index value > 0.6 was considered to have an acceptable predictive value. The multi-factor ROC was utilized for comparisons of the prognostic superior values of the signature and important clinical factors, such as gender, age, stage, grade, and VI. Finally, ICGC and GEO (GSE116174) were chosen as external cohorts to verify the accuracy of our model.

### Nomogram construction

Recently, nomograms have been extensively utilized to predict survival time. In this study, we first used the “rms” package in R to establish a nomogram based on the signature and clinical factors to predict patients’ overall survival. Then, calibration curves were developed to evaluate the accuracy of the nomogram. The 1 -, 2 -, and 3-year ROC curves were used to verify the accuracy of the nomogram.

### Relationship between the SRGs model and the clinical features

The “ggpubr” package was utilized to determine the relationship between the risk score and clinical factors, including the stage, grade, and VI in HCC patients.

### GSEA

To explore the enriched pathways associated with our model, Gene set enrichment analysis (GSEA) was performed using GSEA 4.2.1 software^32^. FDR < 0.05 was considered to indicate statistical significance.

### Association between the signature and immunocytes

We further employed the single-sample gene set enrichment analysis (ssGSEA) in the "gsva" package to assess the difference of 16 immune cells and 13 immune-related pathways in high- and low-risk groups^37^.

### Drug sensitivity prediction

The half-maximal inhibitory concentration (IC50) was applied to explore the association between the risk score and anti-cancer drugs. The IC50 of each HCC sample was predicted using the pRRophetic package in R^38^.

### Statistical analysis

The statistical analysis was used by R software (4.0.2) and Perl language packages. A K–M curve was used to compare the overall survival time of the different groups via the log-rank test. The comparisons between the two groups were analyzed by Wilcoxon rank-sum test. Spearman correlation and Fisher Chi-square tests were performed to measure the correlation between variables. P < 0.05 indicated statistically significant differences.

### Ethics declarations

All methods were carried out by relevant guidelines and regulations.

## Supplementary Information

Supplementary Figure 1.

Supplementary Figure 2.

Supplementary Figure 3.

## Data Availability

The datasets analyzed during the current study are available in public, open access repositories listed in this article. The datasets we analyzed during the current study are available in the TCGA, ICGC and GEO. These datasets can be freely and openly accessed respectively at https://cancergenome.nih.gov/=TCGA; https://dcc.icgc.org and GEO Accession viewer (nih.gov) = GSE116174. The SUMOylation-related genes were downloaded by gene sets “REACTOME_SUMOYLATION” from the GSEA website (https://www.gsea-msigdb.org). The statistical analysis was analyzed using R (version 4.0.2, https://www.R-project.org/).
