# Supplementary material for: A novel prognostic signature for hepatocellular carcinoma based on SUMOylation-related genes
Source: Sci Rep. 2023 Jul 11;13:11233. doi: 10.1038/s41598-023-38197-4 (PMC10336129; doi:10.1038/s41598-023-38197-4)
Supplement: Supplementary file 1 — Supplementary Figure 1. [file 41598_2023_38197_MOESM1_ESM.pdf]

Figure legend：

Supplementary figure 1. Associations between risk score and clinical features. (a) Associations of the risk score and the grade, stage, vascular invasion in TCGA; (b) Association between the stage and the risk score in ICGC.

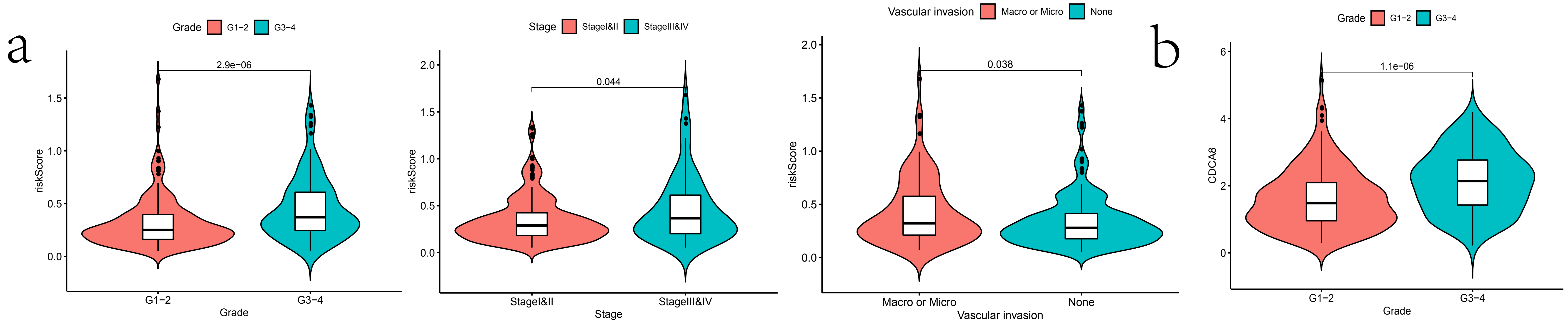

a
Grade G1−2 G3−4
2.9e−06
riskScore
G1−2
G3−4
Grade
Stage StageI&II StageIII&IV
0.044
riskScore
StageI&II
StageIII&IV
Stage
Vascular invasion Macro or Micro None
0.038
riskScore
Macro or Micro
None
Vascular invasion
b
Grade G1−2 G3−4
1.1e−06
CDCA8
G1−2
G3−4
Grade
