# Supplementary material for: A novel prognostic signature for hepatocellular carcinoma based on SUMOylation-related genes
Source: Sci Rep. 2023 Jul 11;13:11233. doi: 10.1038/s41598-023-38197-4 (PMC10336129; doi:10.1038/s41598-023-38197-4)
Supplement: Supplementary file 2 — Supplementary Figure 2. [file 41598_2023_38197_MOESM2_ESM.pdf]

Figure legend：

Supplementary figure 2. The pathways correlated with the high-risk score. The gene set enrichment analysis (GSEA) was performed in the TCGA cohorts to explore underlying mechanisms.

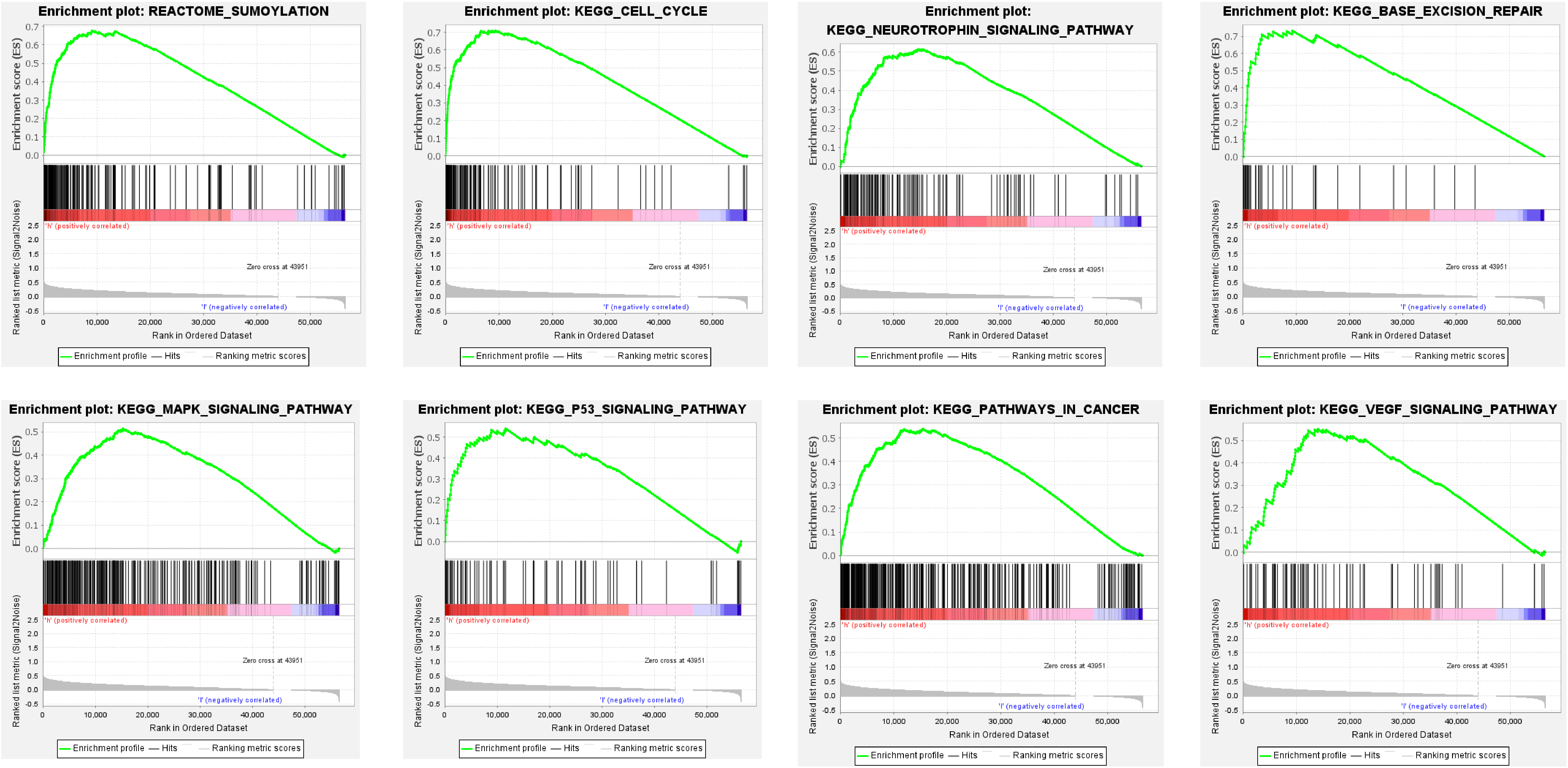

Enrichment plot: REACTOME_SUMOYLATION
Enrichment plot: KEGG_CELL_CYCLE
Enrichment plot: KEGG_NEUROTROPHIN_SIGNALING_PATHWAY
Enrichment plot: KEGG_BASE_EXCISION_REPAIR
Enrichment plot: KEGG_MAPK_SIGNALING_PATHWAY
Enrichment plot: KEGG_P53_SIGNALING_PATHWAY
Enrichment plot: KEGG_PATHWAYS_IN_CANCER
Enrichment plot: KEGG_VEGF_SIGNALING_PATHWAY
Enrichment score (ES)
Ranked list metric (Signal2Noise)
'h' (positively correlated)
'l' (negatively correlated)
Zero cross at 43951
Rank in Ordered Dataset
Enrichment profile
Hits
Ranking metric scores
