# Supplementary material for: A novel prognostic signature for hepatocellular carcinoma based on SUMOylation-related genes
Source: Sci Rep. 2023 Jul 11;13:11233. doi: 10.1038/s41598-023-38197-4 (PMC10336129; doi:10.1038/s41598-023-38197-4)
Supplement: Supplementary file 3 — Supplementary Figure 3. [file 41598_2023_38197_MOESM3_ESM.pdf]

Figure legend：

Supplementary figure 3. The analysis of tumor immune micro-environment. (a,c) Comparison of immune cell abundance and (b,d) immune pathway activation in high- and low-risk groups in the TCGA and ICGC cohort.

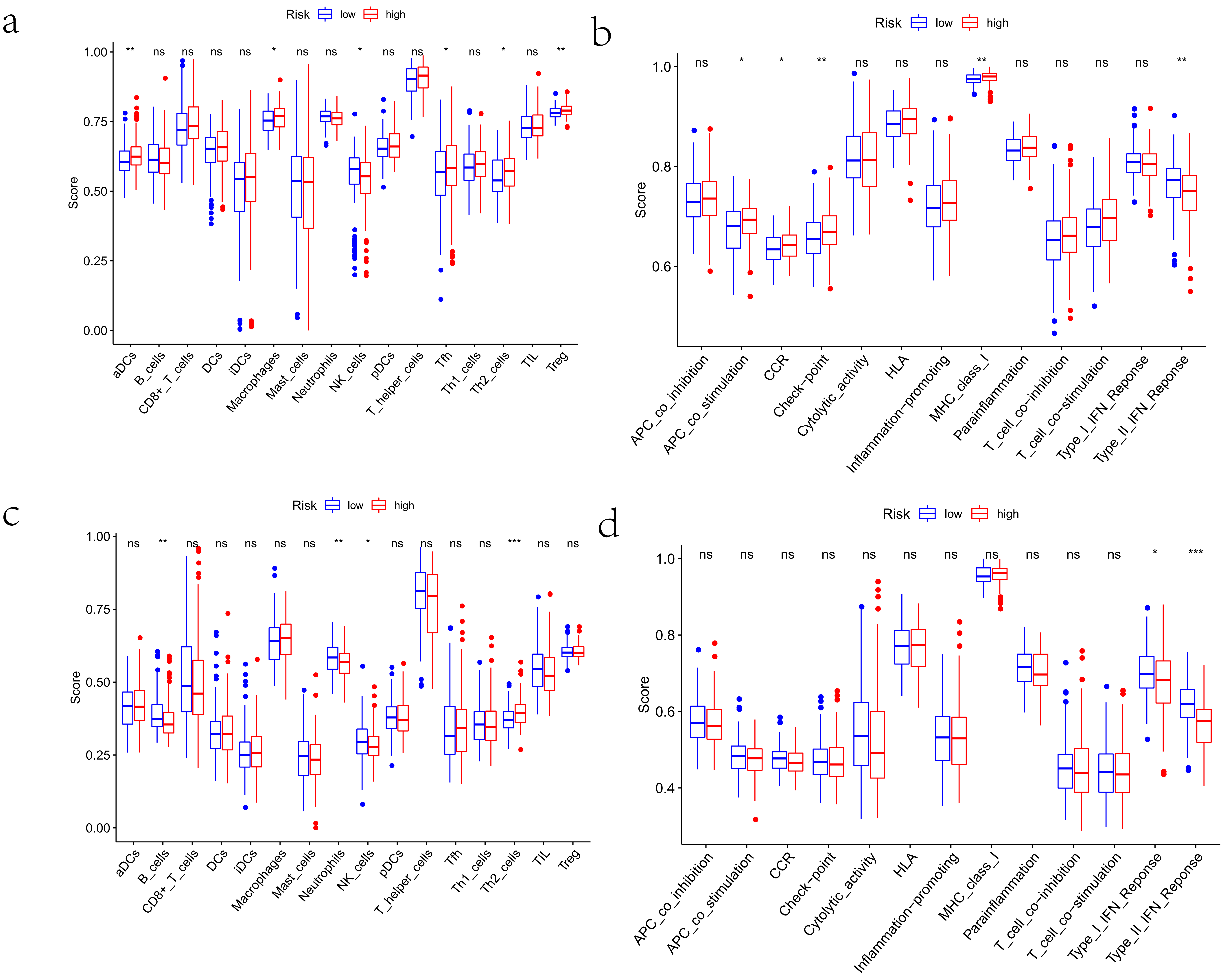
a
Risk low high
Score
1.00
0.75
0.50
0.25
0.00
** ns ns ns ns * ns ns * ns ns * ns * ns **
aDCs
B_cells
CD8+_T_cells
DCs
iDCs
Macrophages
Mast_cells
Neutrophils
NK_cells
pDCs
T_helper_cells
Tfh
Th1_cells
Th2_cells
TIL
Treg
b
Risk low high
Score
1.0
0.8
0.6
ns * * ** ns ns ns ** ns ns ns ns **
APC_co_inhibition
APC_co_stimulation
CCR
Check−point
Cytolytic_activity
HLA
Inflammation−promoting
MHC_class_I
Parainflammation
T_cell_co−inhibition
T_cell_co−stimulation
Type_I_IFN_Reponse
Type_II_IFN_Reponse
c
Risk low high
Score
1.00
0.75
0.50
0.25
0.00
ns ** ns ns ns ns ns ** * ns ns ns ns *** ns ns
aDCs
B_cells
CD8+_T_cells
DCs
iDCs
Macrophages
Mast_cells
Neutrophils
NK_cells
pDCs
T_helper_cells
Tfh
Th1_cells
Th2_cells
TIL
Treg
d
Risk low high
Score
1.0
0.8
0.6
0.4
ns ns ns ns ns ns ns ns ns ns ns * ***
APC_co_inhibition
APC_co_stimulation
CCR
Check−point
Cytolytic_activity
HLA
Inflammation−promoting
MHC_class_I
Parainflammation
T_cell_co−inhibition
T_cell_co−stimulation
Type_I_IFN_Reponse
Type_II_IFN_Reponse
